# Supplementary material for: Breast cancer patient-derived scaffolds enhance the understanding of PD-L1 regulation and T cell cytotoxicity
Source: Commun Biol. 2025 Apr 16;8:621. doi: 10.1038/s42003-025-08054-3 (PMC12003762; doi:10.1038/s42003-025-08054-3)
Supplement: Supplementary file 2 — Supplementary Information [file 42003_2025_8054_MOESM2_ESM.pdf]

Supplementary Information

Breast cancer patient-derived scaffolds enhance the understanding of PD-L1 regulation and T cell cytotoxicity.

Elena Garre [1,2*], Sara Rhost [1], Anna Gustafsson [1], Louis Szeponik [3], Thais Fenz Araujo [1], Marianne Quiding-Järbrink [3], Khalil Helou [4], Anders Ståhlberg [1,5,6] and Göran Landberg [1,2*]

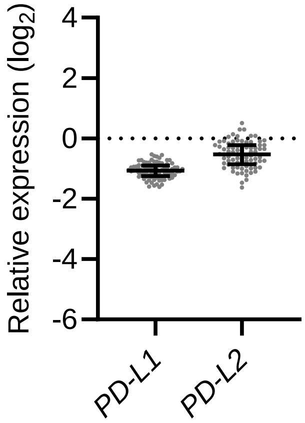

Supplementary Figure 1. Dot plot showing changes in *PD-L1* and *PD-L2* gene expression in patient derived scaffolds (PDS) grown with MDA-MB-231 cancer cells. Individual dots represent relative gene expression of an individual PDS culture (n=84) to gene expression of MDA-MB-231 cultured in 2D (log2-scale). Median and interquartile range (IQR) represented by errors bars are plotted (Supplementary Table 3).

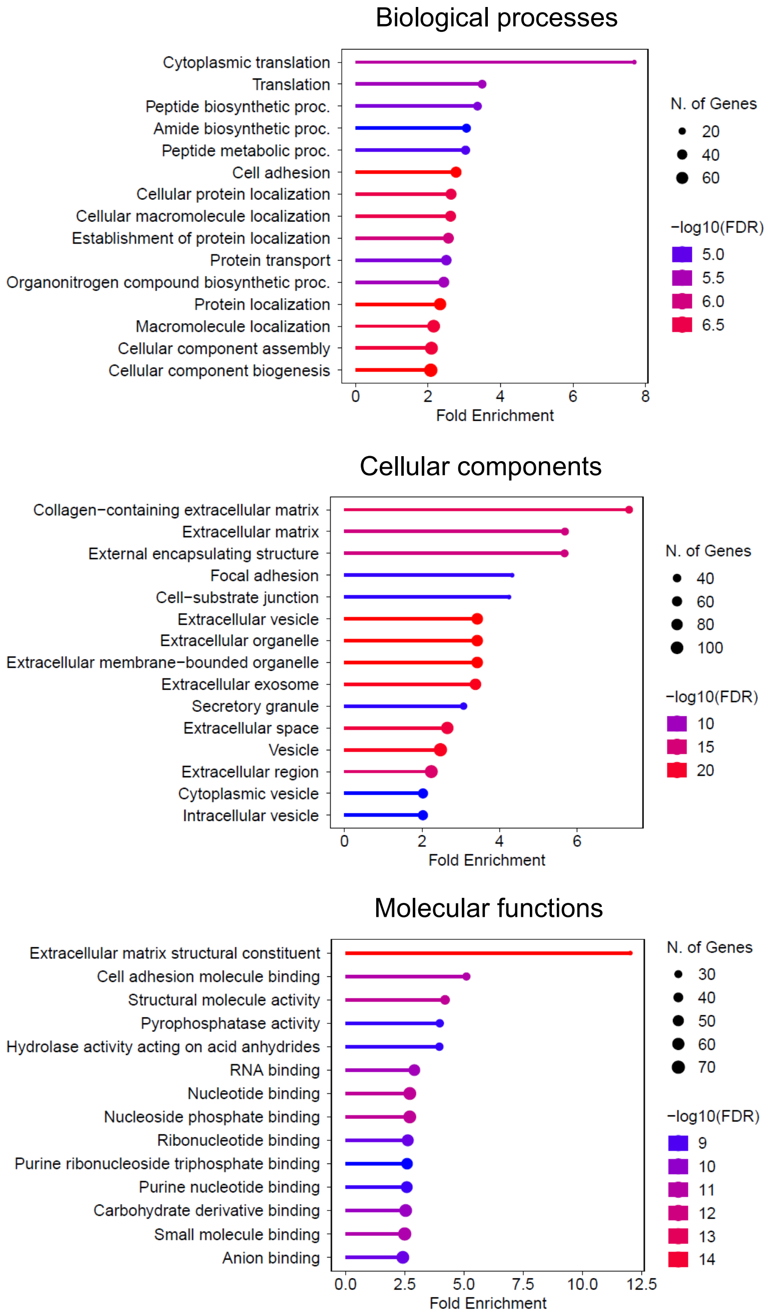

Supplementary Figure 2. Analysis of pathway and process enrichment in the 247 proteins differentially expressed in 59 cell-free PDSs inducing low and high *PD-L1* expression in MCF-7 cancer cells (ShinyGO 0.80, http://bioinformatics.sdstate.edu/go/).

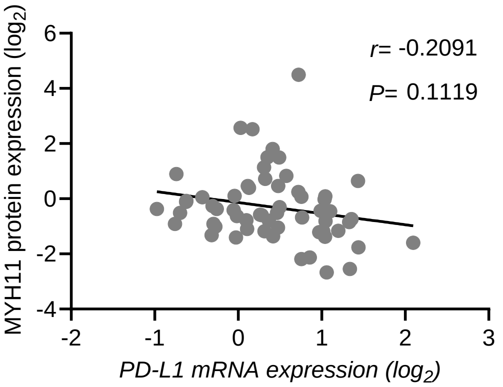

Supplementary Figure 3. Scatter plot illustrating the correlation between MYH11 protein levels and *PD-L1* mRNA levels induced in MCF-7 cancer cells.

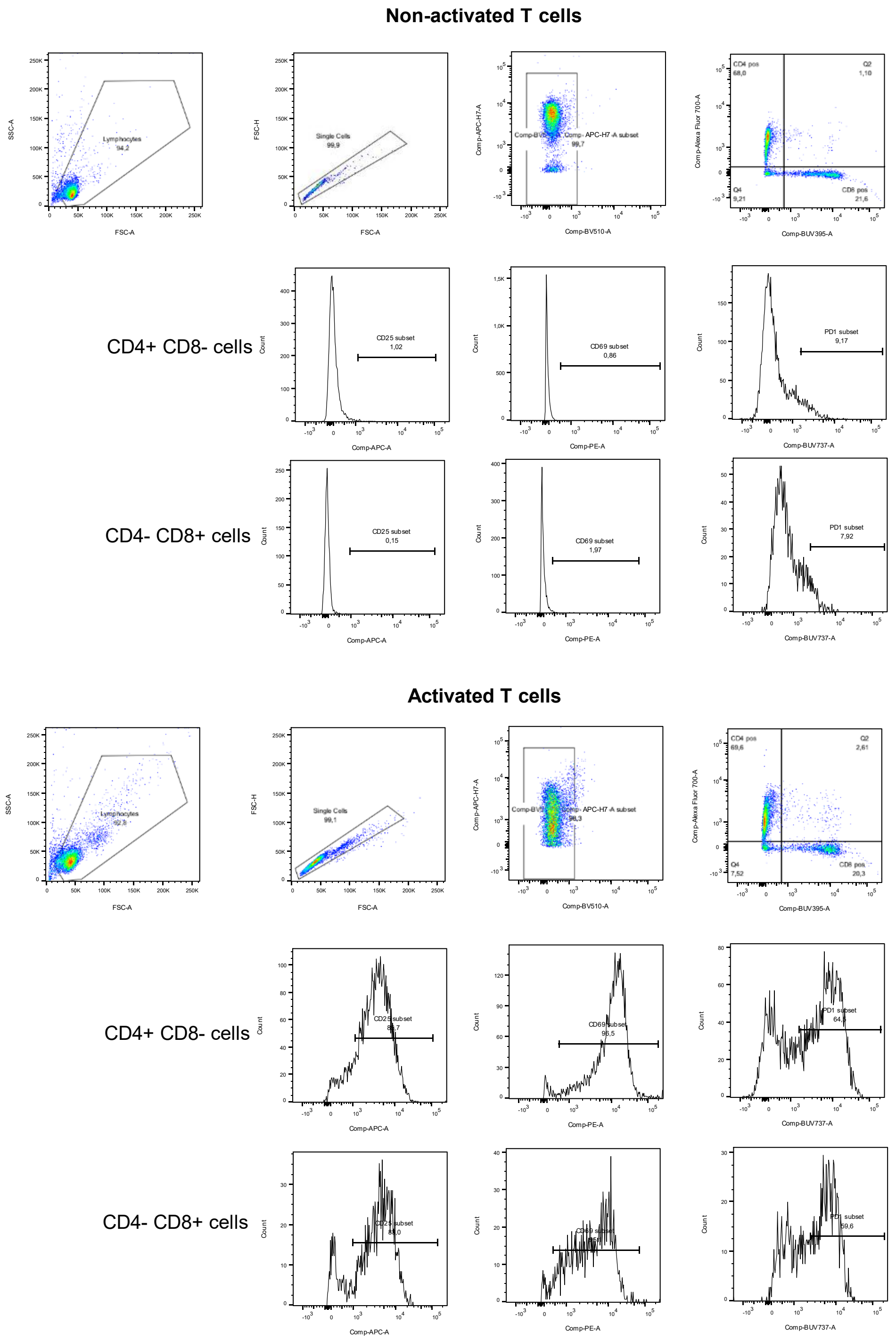

Supplementary Figure 4. Representative FACS-plots depicting the gating strategy for non-activated T cells and strong activated T cells samples, to determine the distribution of activation markers CD25 (APC), CD69 (PE) and PD-1 (BUV737) in $CD4^{+}$ (AF700) and $CD8^{+}$ (BUV395) T cells. (Live/dead cells, BV510; CD3-APC/H7). At least 10 000 alive single cells were analyzed where 98-95% were CD3+. Gating strategy: 1. Exclude cell debris and other cell types: SSC-A/FSC-A. 2. Exclude doublets. Single cell gate: FSC-H/FSC-A. 3. Select alive cells expression CD3+: CD3-APC-H7/BV510-A (live/dead stain).

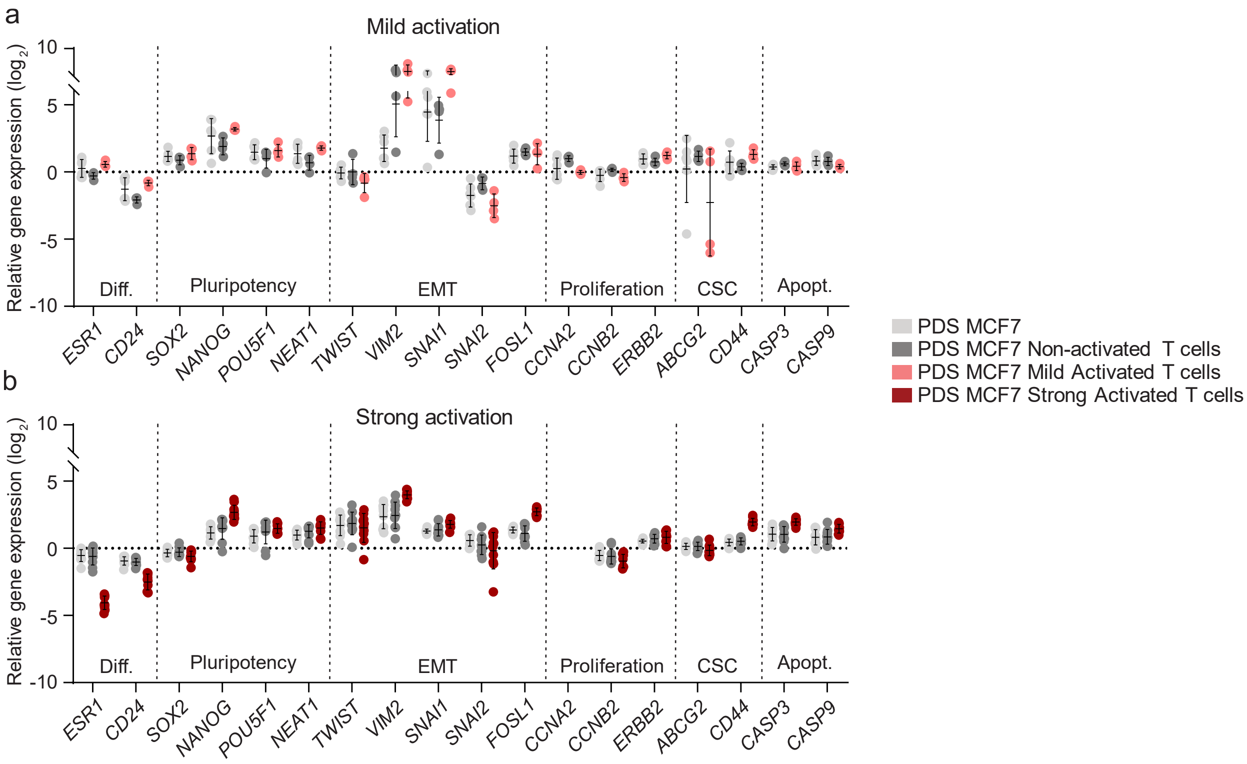

Supplementary Figure 5. Relative expression of genes associated to cancer processes (differentiation – Diff., pluripotency, epithelial-to-mesenchymal transition – EMT, proliferation, cancer stem cell - CSC; apoptosis – Apt.) in MCF-7 PDS monocultures or co-cultures with non-activated T cells, mild activated T cells and strong activated T cells. Average and standard deviation is plotted (replicates=4-10). Gene expression is expressed relative to 2D MCF-7 cultures and in log2-scale.

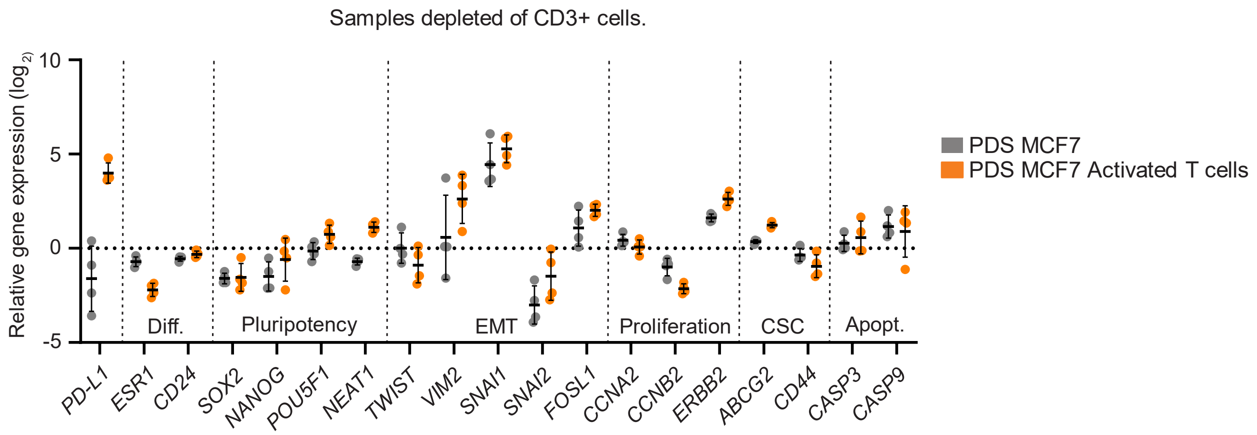

Supplementary Figure 6. Relative expression of genes associated to cancer processes (differentiation – Diff., pluripotency, epithelial-to-mesenchymal transition – EMT, proliferation, cancer stem cell - CSC; apoptosis – Apt.) in MCF-7 PDS monocultures and co-cultures with strong activated T cells where T cells were depleted from the cell sample immediately before RNA extraction using Dynabeads^{TM} CD3 (Invitrogen). Average and standard deviation is plotted (replicates=4). Gene expression is expressed relative to 2D MCF-7 cultures and in log2-scale.

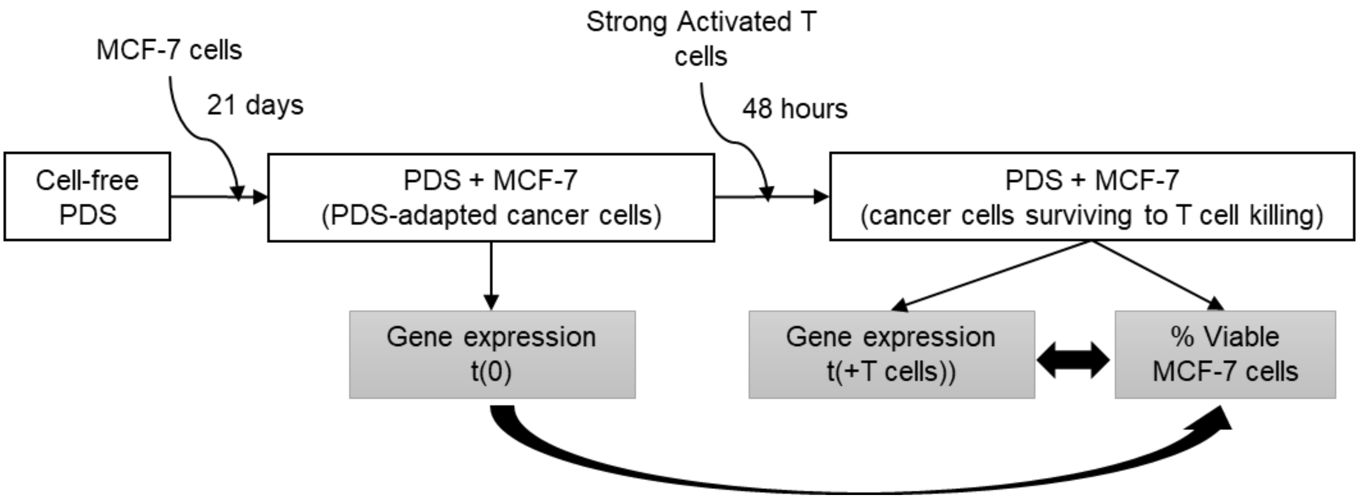

Supplementary Figure 7. Schema depicting the experimental workflow for MCF-7 PDS co-cultures with active T cells, and the influence of the cancer cell adaptation to PDSs into the subsequent response to T cells.

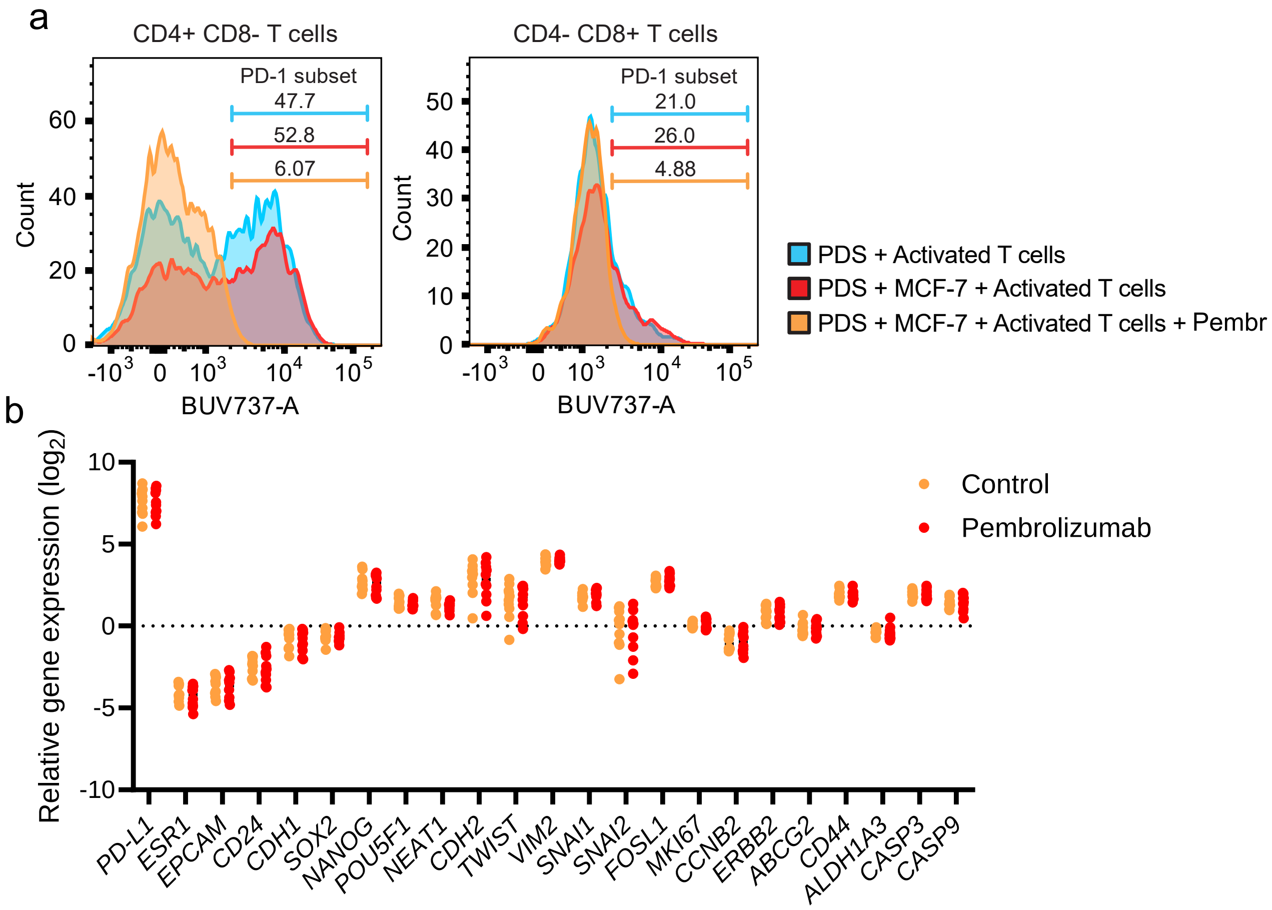

Supplementary Figure 8. a) Pembrolizumab (Pembr) PD-1 receptor blocking efficiency in PDS co-cultures of MCF-7 cancer cells and strongly activated T cells indicated by percentage of $CD3^+CD4^+CD8^-$ or $CD3^+CD4^-CD8^+$ cell subsets expressing PD-1 marker (BUV737). b) Relative expression of *PD-L1* and genes associated to cancer processes (differentiation, pluripotency, EMT, proliferation, CSC and apoptosis) in MCF-7 PDS co-cultures with strongly activated T cells treated or not with 100 µg mL^{-1} Pembrolizumab. Each dot represents an individual PDS culture. Gene expression is expressed relative to 2D MCF-7 cultures and in log2-scale.

Supplementary Table 1. Clinical information. Baseline characteristics and distribution of the patients involved in the study using patient-derived scaffolds (PDSs) cultured with either MCF-7 or MDA-MB-231. Total number of patients=110.

	PDSs cultured with MCF-7	PDSs cultures with MDA-MB-231	PDS used for MS
**Number of patients, n**	110	84	59
**Age at diagnosis, years**			
Median (range)	65 (27-88)	66 (27-88)	69 (43-86)
**Menopausal status, n (%)**			
Premenopausal	26 (23.6)	22 (26.2)	11 (18.6)
Postmenopausal	76 (69.1)	55 (65.5)	44 (74.6)
Unknown	8 (7.3)	7 (8.3)	4 (6.8)
**Estrogen receptor status, n (%)**			
Negative	19 (17.3)	15 (17.9)	9 (15.3)
Positive	89 (80.9)	67 (79.8)	49 (83.1)
Unknown	2 (1.8)	2 (2.3)	1 (1.7)
**Progesterone receptor, n (%)**			
Negative	35 (31.8)	25 (29.8)	19 (32.2)
Positive	72 (65.5)	56 (66.7)	38 (64.4)
Unknown	3 (2.7)	3 (3.5)	2 (3.4)
**Histological grade, n (%)**			
1	28 (25.4)	23 (27.4)	18 (30.5)
2	39 (35.5)	27 (32.1)	16 (27.1)
3	41 (37.3)	32 (38.2)	24 (40.7)
Unknown	2 (1.8)	2 (2.3)	1 (1.7)
**Tumor size, mm**			
Median (range)	30 (6-90)	30 (6-90)	30 (6-90)
**Cancer type, n (%)**			
Ductal	77 (70.0)	61 (72.6)	38 (64.4)
Lobular	20 (18.2)	14 (16.7)	16 (27.1)
Other	11 (10.0)	8 (9.5)	5 (8.5)
Unknown	2 (1.8)	1 (1.2)	0 (0.0)
**Lymph node metastasis, n (%)**			
No	38 (34.5)	29 (34.5)	20 (33.9)
Yes	54 (49.1)	42 (50.0)	29 (49.2)
Unknown	18 (16.4)	13 (15.5)	10 (16.9)
**Disease free survival, n (%)**			
No recurrence, alive or death by other cause	39 (35.5)	30 (35.7)	20 (33.9)
Recurrence or death by cancer	51 (46.4)	39 (46.4)	28 (47.5)
Unknown	20 (18.1)	15 (17.9)	11 (18.6)
**Follow up, months**			
Median (range)	88 (1-200)	95 (1-200)	95 (1-192)

Supplementary Table 2. *PD-L1* and *PD-L2* expression levels in 2D cultures of MCF-7 or MDA-MB-231 (Cq values). Different passages were quantified.

	Cq values	
	PD-L1	*PD-L2*
2D_MCF7_P22_1	30.42	38.14
2D_MCF7_P22_2	29.13	
2D_MCF7_P23_1	28.36	
2D_MCF7_P23_2	28.53	
2D_MCF7_P28	28.17	
2D_MCF7_P29	28.43	
2D_231_P2	24.58	27.46
2D_231_P3	24.66	27.70
2D_231_P14	24.98	28.19
2D_231_P20	24.11	27.41
2D_231_P23	24.53	28.21

Supplementary Table 3. *PD-L1* and *PD-L2* expression median and interquartile range in MCF-7 PDS cultures.

	MCF-7		MDA-MB-231	
	Median	IQR	Median	IQR
PD-L1	0.49	0.822	-1.067	0.352
PD-L2	3.2	0.7	-0.528	0.636

Supplementary Table 4. Associations between changes in *PD-L1* and *PD-L2* gene expression and clinico-pathological features. Data was analyzed using SPSS statistics (IBM). Non-parametric Mann-Whitney U statistical test was performed for assessment of clinico-pathological variables relationship with individual gene expression in PDSs. Number of cases (n) and median are included. Kaplan Meier cut-offs for disease-free survival are shown and Log-rank statistical test was applied to compare survival in different strata (M, median; Q1, first quartile; Q3, third quartile), and the p-value are shown in this table. P-values <0.05 were considered significant.

	Grade					Estrogen receptor (ER)				
	Low		High			ER+		ER-		
All PDS MCF-7 (n=110)	n	Median	n	Median	p-value	n	Median	n	Median	p-value
PD-L1	60	0.35	40	0.76	0.001	81	0.49	19	0.57	0.867
PD-L2	-	-	-	-	-	-	-	-	-	-

	Progesterone receptor (PR)					Cancer type				
	PR+		PR-			lobular		ductal		
All PDS MCF-7 (n=110)	n	Median	n	Median	p-value	n	Median	n	Median	p-value
PD-L1	64	0.475	35	0.72	0.221	19	0.11	71	0.54	0.024
PD-L2	-	-	-	-	-	-	-	-	-	-

	Lymph node metastasis (LN Met)					Recurrence				
	No		Yes			No		Yes		
All PDS MCF-7 (n=110)	n	Median	n	Median	p-value	n	Median	n	Median	p-value
PD-L1	51	0.42	55	0.539	0.677	55	0.53	46	0.42	0.813
PD-L2	-	-	-	-	-	-	-	-	-	-

	Disease-free survival (DFS)		
	Kaplan_Meier (Log Rank)		
All PDS MCF-7 (n=110)	Median	Q1	Q3
PD-L1	0.656	0.122	0.091
PD-L2	-	-	-

	Grade					Estrogen receptor (ER)				
	Low		High			ER+		ER-		
All PDS MDA-MB-231 (n=84)	n	Median	n	Median	p-value	n	Median	n	Median	p-value
PD-L1	50	-1.063	33	-1.089	0.921	68	-1.049	15	-1.165	0.169
PD-L2	49	-0.575	29	0.415	0.151	65	-0.448	13	-0.621	0.713

	Progesterone receptor (PR)					Cancer type				
	PR+		PR-			lobular		ductal		
All PDS MDA-MB-231 (n=84)	n	Median	n	Median	p-value	n	Median	n	Median	p-value
PD-L1	57	-1.045	25	-1.119	0.121	15	-0.979	61	-1.079	0.520
PD-L2	54	-0.453	23	-0.528	0.396	14	-0.738	57	-0.528	0.439

	Lymph node metastasis (LN Met)					Recurrence				
	No		Yes			No		Yes		
All PDS MDA-MB-231 (n=84)	n	Median	n	Median	p-value	n	Median	n	Median	p-value
PD-L1	29	-1.029	43	-1.089	0.112	44	-1.049	40	-1.103	0.336
PD-L2	29	-0.528	38	-0.636	0.173	40	-0.446	39	-0.631	0.219

	Disease-free survival (DFS)		
	Kaplan_Meier (Log Rank)		
All PDS MDA-MB-231 (n=84)	Median	Q1	Q3
PD-L1	0.411	0.806	0.374
PD-L2	0.381	0.165	0.831

Supplementary Table 5. Spearman correlations of *PD-L1* and *PD-L2* expression with breast cancer processes markers (EMT, proliferation, CSC and apoptosis) in PDSs cultured with MCF-7. p<0.05 were considered significant[23].

	MCF-7	
rs-values	*PD-L1*	*PD-L2*
SOX2	-0.233	0.168
NANOG	0.015	0.057
POU5F1	0.09	-0.089
NEAT1	-0.048	0.079
MKI67	-0.036	-0.246
CCNA2	-0.122	-0.018
CCNB2	-0.178	-0.496
ERBB2	-0.098	-0.075
VIM	0.136	0.164
SNAI1	-0.176	-0.125
SNAI2	**0.415**	0.321
FOSL1	0.013	-0.282
CD44	-0.02	0.375
ABCG2	-0.195	-0.464
ALDH1A3	0.315	0.091
CASP9	0.076	0.068
CASP3	0.171	0.093

	MCF-7	
p-values	*PD-L1*	*PD-L2*
SOX2	**0.017**	0.55
NANOG	0.883	0.84
POU5F1	0.364	0.751
NEAT1	0.626	0.781
MKI67	0.719	0.376
CCNA2	0.218	0.95
CCNB2	0.071	0.06
ERBB2	0.321	0.791
VIM	0.168	0.558
SNAI1	0.076	0.657
SNAI2	**0.001**	0.243
FOSL1	0.893	0.308
CD44	0.844	0.168
ABCG2	0.048	0.081
ALDH1A3	**0.001**	0.747
CASP9	0.441	0.81
CASP3	0.083	0.742

Supplementary Table 6. Median and interquartile range (IQR) for the % alive MCF-7/Luciferase cells, in PDS co-cultures with non-activated and activated T cells.

		% alive MCF-7/Luc cells			
		2D MCF-7		PDS MCF-7	
		Median	IQR	Median	IQR
Non-activated T cells	48 hours	96.7	14.7	82.7	39.6
	72 hours	97.5	16.7	68.3	37.2
Activated T cells	Mild	100.3	67.2	53.0	27.1
	Strong	3.0	1.8	27.1	21.0

Supplementary Table 7. Median and interquartile range (IQR) for the *PD-L1* expression in PDS co-cultures with non-activated and activated T cells.

		PD-L1 expression							
		2D MCF-7 w/o T cells		2D MCF-7 + T cells		PDS MCF-7 w/o T cells		PDS MCF-7 + T cells	
		Median	IQR	Median	IQR	Median	IQR	Median	IQR
Non-activated T cells	48 hours	-0.27	0.74	1.08	2.30	1.00	0.53	1.83	0.94
	72 hours	0.07	0.26	0.78	1.21	1.07	1.54	2.19	0.63
Activated T cells	Mild	0.11	0.82	3.50	2.03	1.71	0.70	7.87	0.16
	Strong	-0.27	0.74	8.06	0.40	1.00	0.53	7.65	0.94

Supplementary Table 8. Spearman correlations (p-value) between *PD-L1* expression and % alive MCF-7/Luciferase cancer cells in PDS co-cultures with non-activated or strong-activated T cells. (p<0.05 were considered significant).

	PD-L1 t0	PD-L1 (+Non activate T cells)	PD-L1 (+Strong Activated T cells)	% MCF-7/Luc (+Non-activated T cells)	% MCF-7/Luc (+Strong activated T cells)
PD-L1 t0	-				
PD-L1 (+Non activate T cells)	0.112	-			
PD-L1 (+Strong Activated T cells)	0.471	0.002	-		
% MCF-7/Luc (+Non-activated T cells)	0.762	0.915	0.859	-	
% MCF-7/Luc (+Strong activated T cells)	0.174	**0.02**	0.071	**0.029**	-

Supplementary Table 9. Clinical information. Baseline characteristics and distribution of the patients involved in PDS co-cultures of MCF-7 with mild and strong activated T cells.

	PDSs MCF-7 and Mild T cells	PDSs MCF-7 and Strong Activated T cells
**Number of patients, n**	5	12
**Estrogen receptor status, n**		
Negative	1	2
Positive	4	10
**Progesterone receptor, n**		
Negative	3	9
Positive	2	3
**HER2 receptor, n**		
Negative	4	9
Positive	1	3
**Histological grade, n**		
1	0	0
2	1	2
3	2	7
Unknown	2	3
**Cancer type, n**		
Ductal	4	8
Lobular	1	4
**TILS %, n**		
1-19%	4	6
10-49%	1	3
50-74%	0	1
Unknown	0	2
**Ki67 %**		
Median (range)	22 (9-58)	26 (11-95)

Supplementary Table 10. *PD-L1* expression and cancer cell viability associations to clinical features in PDS co-cultures. P-values of Mann-Whitney U test (Type, Grade, ER, PR, HER2), Kruskal-Wallis H test (TILS%) and Spearman and Pearson correlations (Ki67%) for *PD-L1* expression and % alive MCF-7/Luciferase cells in PDS co-cultures with T cells (non- and strong-activated) and clinical parameters. Cancer type (ductal versus lobular), grade (low II versus high III), estrogen receptor (ER; positive versus negative), progesterone receptor (PR; positive versus negative), tumor infiltrated T cells (TILS; 1-9%, 10-49%, 50-74%), HER2 receptor (positive versus negative). ($p<0.05$ were considered significant).

							Ki67 %	
	Type	Grade	ER	PR	TILS %	HER2	Spearman	Pearson
PD-L1 t0	0.734	0.77	0.197	0.405	0.782	0.309	0.191	0.376
PD-L1 (+Non activate T cells)	0.61	0.558	0.133	0.872	0.229	0.405	0.241	0.095
PD-L1 (+Strong Activated T cells)	0.865	0.242	0.519	0.116	0.229	0.405	0.694	0.992
% MCF-7/Luc (+Non-activated T cells)	0.089	0.143	0.283	0.309	0.849	0.405	0.266	0.317
% MCF-7/Luc (+Strong activated T cells)	0.12	0.766	0.063	0.51	0.676	0.778	0.067	**0.044**

Supplementary Table 11. Correlations of cancer cell viability with gene expression in PDS-co-cultures. Spearman correlations (rs) of % alive MCF-7/Luciferase cancer cells with the expression of breast cancer processes markers (EMT, proliferation, CSC and apoptosis) in PDSs co-cultured with strong activated T cells. rs>|0.5| and p<0.05 were considered significant. t(0), MCF-7/Luciferase gene expression adapted to the PDS before adding T cells. t (+T cells). MCF-7/Luciferase gene expression adapted to the PDS after incubating 48 hours with strong activated T cells.

rs-values	t(0)	t(+T cells)
PD-L1	-0.401	-0.515
ESR1	-0.022	0.134
CD24	0.164	-0.047
CDH1	-0.067	**-0.632**
SOX2	0.326	-0.465
NANOG	**-0.563**	0.131
POU5F1	-0.156	0.384
NEAT1	-0.192	0.41
CDH2	0.436	**0.669**
TWIST	0.451	**0.713**
VIM2	0.435	**0.786**
SNAI1	**0.735**	0.074
SNAI2	0.153	0.485
FOSL1	-0.078	**-0.691**
MKI67	-0.62	0.018
CCNB2	0.304	0.426
ERBB2	0.412	-0.267
ABCG2	0.359	**0.655**
CD44	**0.68**	0.167
ALDH1A3	**0.56**	0.033
CASP3	0.437	0.362
CASP9	0.465	**0.674**

p-values	t(0)	t(+T cells)
PD-L1	0.174	0.071
ESR1	0.942	0.663
CD24	0.592	0.878
CDH1	0.853	**0.05**
SOX2	0.277	0.109
NANOG	**0.045**	0.669
POU5F1	0.611	0.195
NEAT1	0.529	0.165
CDH2	0.208	**0.034**
TWIST	0.122	**0.006**
VIM2	0.138	**0.001**
SNAI1	**0.004**	0.839
SNAI2	0.672	0.156
FOSL1	0.8	**0.009**
MKI67	0.056	0.96
CCNB2	0.312	0.146
ERBB2	0.162	0.377
ABCG2	0.228	**0.015**
CD44	**0.011**	0.585
ALDH1A3	**0.047**	0.914
CASP3	0.135	0.224
CASP9	0.109	**0.012**

Supplementary Table 12. Associations between gene expression and Pembrolizumab treatment in PDS co-cultures. P-values of Mann-Whitney U test for PDS responding or not responding to pembrolizumab and the expression of breast cancer processes markers (EMT, proliferation, CSC and apoptosis) in the surviving MCF-7/Luciferase cancer cells. ($p<0.05$ were considered significant).

	Responder/Non-responder PDS
PD-L1	0.917
ESR1	0.754
EPCAM	0.754
CD24	0.602
CDH1	0.917
SOX2	**0.016**
NANOG	0.917
POU5F1	0.917
NEAT1	0.175
CDH2	0.754
TWIST	0.602
VIM2	0.917
SNAI1	0.753
SNAI2	0.347
FOLS1	0.116
MKI67	**0.028**
CCNB2	0.465
ERBB2	0.917
ABCG2	0.754
CD44	0.059
ALDH1A3	0.527
CASP3	0.175
CASP9	0.754

Supplementary Table 13. Primers. List of genes and additional information. EMT = Epithelial-mesenchymal transition. CSC = cancer stem cells. BCSC = Breast cancer stem cells.

Gene	Category	Ensembl Gene ID	Forward Primer [5'-3']	Reverse Primer [5'-3']
ABCG2	BCSC	ENSG00000118777	GGTGGAGGCAAATCTTCGTTA	GAGTGCCCATCACAACATCA
ALDH1A3	BCSC	ENSG00000184254	AAAAAGAGCGAATAGCACCG	GCATAGAGGGCGTTGTAGCA
CASP3	Apoptosis	ENSG00000164305	GGCGTGTCATAAAATACCAGTG	GCGTCAAAGGAAAAGGACTCAAA
CASP9	Apoptosis	ENSG00000132906	GGTGATGTCGGTGCTCTTGA	TTTCTTGGCAGTCAGGTCGC
CCNA2	Proliferation	ENSG00000145386	AAGACGAGACGGGTTGC	GGCTGTTTACTGTTTGCTTTCC
CCNB2	Proliferation	ENST00000288207	CGACCCTTGCCACTACACTT	TGACTTCCAATACTTCATTCTCTG
CD24	Differentiation	ENSG00000272398	GCTCCTACCCACGCAGATT	GGTGGTGGCATTAGTTGGAT
CD44	BCSC	ENSG00000026508	GAAGAAGGTGTGGGCAGAAGA	ACCATTTCCTGAGACTTGCTG
CDH1	Differentiation	ENSG00000039068	AGAGGACCAGGACTTTGACTTG	CAGAGAATCATAAGGCGGGG
CDH2	EMT	ENSG00000170558	CATTATCAACCCCATCTCGG	ACTGTCCCATTCCAAACCTG
ERBB2	Differentiation	ENST00000443427	TGACTATGCTTCAGGCTACCAT	ACCTTTCATCATTCCCACTTC
ESR1	EMT	ENSG00000175592	GCAGGCGGAGACTGACAA	GGGGAAAGGGAGATACAAGG
FOSL1	EMT	ENSG00000175592	GCAGGCGGAGACTGACAA	GGGGAAAGGGAGATACAAGG
MKI67	Proliferation	ENSG00000148773	TGGGTCTGTTATTGATGAGCC	CATCAGGGTCAGAAGAGAAGC
NANOG	Pluripotency	ENSG00000111704	CCTATGCCTGTGATTTGTGG	AAGTGGGTTGTTTGCCTTTG
NEAT1	Pluripotency	ENSG00000245532	GCCTTCTTGTGCGTTTCTCG	CCCTCCCAGCGTTTAGC
PD-L1	Immune	ENSG00000120217	GCTGAACGCATTTACTGTCACG	CCAGGGAGAGCTGGTCCTTC
PD-L2	Immune	ENSG00000197646	GCTTCACATTTTCATCCCCTTCT	GGTTCAGATAGCACTGTTCACTT
POU5F1	Pluripotency	ENSG00000204531	CGAAAGAGAAAGCGAACCAG	AACCACACTCGGACCACATC
SNAI1	EMT	ENSG00000124216	TAATCCAGAGTTTACCTTCCAGCA	AGCCTTTCCCACTGTCCTCA
SNAI2	EMT	ENSG00000019549	GCCAAACTACAGCGAACTGG	AGGAGGTGTCAGATGGAGGA
SOX2	Pluripotency	ENSG00000181449	ACACCAATCCCATCCACACT	CCTCCCCAGGTTTTCTCTGT
TWIST	EMT	ENST00000242261	GGACAGTGATTCCCAGACGG	CATAGTGATGCCTTTCCTTTCAG
VIM2	EMT	ENSG00000026025	CAGATGCGTGAAATGGAAGA	TGGAAGAGGCAGAGAAATCC
EIF1	Housekeeping	ENSG00000173812	TCGTATGTCCGCTATCCAGA	TAAGGGTCTTCCTGCCGTTT
GAPDH	Housekeeping	ENSG00000111640	AGTCAGCCGCATCTTCTTTT	CGCCCAATACGACCAAAT
RPS10	Housekeeping	ENSG00000124614	AGCCGCAGAGATGTTGATG	CCTCGGGACTTGAGAGACTG
RPS26	Housekeeping	ENSG00000197728	GATGCGTGCCCAAGGAC	CAGGTCTAAATCGGGGTGG
YWHAZ	Housekeeping	ENSG00000164924	ACGCCTCACTCCCGTTT	CTGGATGTTCTGCTGGCTC
