# Supplementary material for: Breast cancer patient-derived scaffolds enhance the understanding of PD-L1 regulation and T cell cytotoxicity
Source: Commun Biol. 2025 Apr 16;8:621. doi: 10.1038/s42003-025-08054-3 (PMC12003762; doi:10.1038/s42003-025-08054-3)
Supplement: Supplementary file 3 — Description of Additional Supplementary Materials [file 42003_2025_8054_MOESM3_ESM.pdf]

Description of Additional Supplementary Files

File name: Supplementary Data 1

Description: Proteins significantly differentially expressed (247) in cell-free PDSs. Classified by their capacity of inducing high or low PD-L1 expression when cultured with MCF-7 cancer cells. The 10 most significant proteins are highlighted (orange) and summary description included (https://www.proteinatlas.org/).

File name: Supplementary Data 2

Description: Numerical source data for graphs
