# Supplementary material for: Breast cancer patient-derived scaffolds enhance the understanding of PD-L1 regulation and T cell cytotoxicity
Source: Commun Biol. 2025 Apr 16;8:621. doi: 10.1038/s42003-025-08054-3 (PMC12003762; doi:10.1038/s42003-025-08054-3)
Supplement: Supplementary file 6 — Reporting Summary [file 42003_2025_8054_MOESM6_ESM.pdf]

nature portfolio

Corresponding author(s): Elena Garre
Göran Landberg

Last updated by author(s): Apr 7, 2025

# Reporting Summary

Nature Portfolio wishes to improve the reproducibility of the work that we publish. This form provides structure for consistency and transparency in reporting. For further information on Nature Portfolio policies, see our Editorial Policies and the Editorial Policy Checklist.

## Statistics

For all statistical analyses, confirm that the following items are present in the figure legend, table legend, main text, or Methods section.

| n/a | Confirmed | |
|---|---|---|
| ☐ | ☒ | The exact sample size (*n*) for each experimental group/condition, given as a discrete number and unit of measurement |
| ☐ | ☒ | A statement on whether measurements were taken from distinct samples or whether the same sample was measured repeatedly |
| ☐ | ☒ | The statistical test(s) used AND whether they are one- or two-sided<br>*Only common tests should be described solely by name; describe more complex techniques in the Methods section.* |
| ☒ | ☐ | A description of all covariates tested |
| ☒ | ☐ | A description of any assumptions or corrections, such as tests of normality and adjustment for multiple comparisons |
| ☐ | ☒ | A full description of the statistical parameters including central tendency (e.g. means) or other basic estimates (e.g. regression coefficient) AND variation (e.g. standard deviation) or associated estimates of uncertainty (e.g. confidence intervals) |
| ☐ | ☒ | For null hypothesis testing, the test statistic (e.g. *F*, *t*, *r*) with confidence intervals, effect sizes, degrees of freedom and *P* value noted<br>*Give P values as exact values whenever suitable.* |
| ☒ | ☐ | For Bayesian analysis, information on the choice of priors and Markov chain Monte Carlo settings |
| ☒ | ☐ | For hierarchical and complex designs, identification of the appropriate level for tests and full reporting of outcomes |
| ☐ | ☒ | Estimates of effect sizes (e.g. Cohen's *d*, Pearson's *r*), indicating how they were calculated |

*Our web collection on statistics for biologists contains articles on many of the points above.*

## Software and code

Policy information about availability of computer code

| | |
|---|---|
| Data collection | No software was used |
| Data analysis | - Gene expression analyses: GenEx (MultiId)<br>- Statistical analyses and visualization: SPSS statistics v.25 (IBM) and Prism 10 (GraphPad)<br>- Flow cytometry data analyses: FlowJo software (TreeStar Inc)<br>- Pathways and processes enrichment of mass spectrometry data: ShinyGO 0.80 (http://bioinformatics.sdstate.edu/go/).<br>- Enrichment for transcription factor targets of mass spectrometry data TRRUST database and Metascape (https://metascape.org). |

For manuscripts utilizing custom algorithms or software that are central to the research but not yet described in published literature, software must be made available to editors and reviewers. We strongly encourage code deposition in a community repository (e.g. GitHub). See the Nature Portfolio guidelines for submitting code & software for further information.

## Data

Policy information about availability of data

All manuscripts must include a data availability statement. This statement should provide the following information, where applicable:

- Accession codes, unique identifiers, or web links for publicly available datasets
- A description of any restrictions on data availability
- For clinical datasets or third party data, please ensure that the statement adheres to our policy

The data supporting the findings of this study are available within the paper, Supplementary Information and Supplementary Data 1 and 2 files. The mass spectrometry data that support the findings have been deposited to the ProteomeXchange Consortium (https://www.proteomexchange.org) via the PRIDE partner repository with the dataset identifier PXD042671.

## Research involving human participants, their data, or biological material

Policy information about studies with human participants or human data. See also policy information about sex, gender (identity/presentation), and sexual orientation and race, ethnicity and racism.

| | |
|---|---|
| Reporting on sex and gender | Only sex (biological attribute) was considered in the study design and only female individuals were included. Sex and gender-based analyses are not included in this study because it is only focused in female breast cancer. |
| Reporting on race, ethnicity, or other socially relevant groupings | These variables are not relevant for our study. |
| Population characteristics | Females between 22-88 years diagnosed with primary breast cancer and without neoadjuvant treatment. |
| Recruitment | Participants were recruited from female patients diagnosed with breast cancer before the primary surgery at Sahlgrenska University Hospital (Gothenburg, Sweden). |
| Ethics oversight | Regional Research Ethics Committee (Regionala Etikprövningsnämnden) in Gothenburg (DNR: 515-12, S164-02 and T972-18) |

Note that full information on the approval of the study protocol must also be provided in the manuscript.

# Field-specific reporting

Please select the one below that is the best fit for your research. If you are not sure, read the appropriate sections before making your selection.

☒ Life sciences ☐ Behavioural & social sciences ☐ Ecological, evolutionary & environmental sciences

For a reference copy of the document with all sections, see nature.com/documents/nr-reporting-summary-flat.pdf

# Life sciences study design

All studies must disclose on these points even when the disclosure is negative.

| | |
|---|---|
| Sample size | Samples sizes used for biobanked tumors (MCF7 cultures, n=110; MDA-MB-23 cultures, n= 84; mass spectrometry, n=59) were established in our previous published studies where the statistical power was already proved. For tumors gathered directly after surgery, at least 5 were included in each group. |
| Data exclusions | No data was excluded from the analyses. |
| Replication | Replication was successful and all the attempts (replicates) are shown in the figures, despite that there are data deviation due the tumor heterogeneity between patients and also between T cells healthy donors. |
| Randomization | The selection of the Biobanked tumors was not random, we selected samples representative of the proportions for each clinical variable in the global statistics for women diagnosed with breast cancer. We included the maximum number of tumors based in sample availability for the PDS cultures with different cell lines and mass spectrometry. For tumors collected directly after surgery, they were randomly allocated in the experiments based in the availability after the surgery at the clinical pathology diagnostic unit. |
| Blinding | This was not relevant to our study. The investigators were not blinded but the clinical information was not used during data collection and processing (gene expression, protein levels, viability measurements) till association analyses were performed. |

# Behavioural & social sciences study design

All studies must disclose on these points even when the disclosure is negative.

| | |
|---|---|
| Study description | *Briefly describe the study type including whether data are quantitative, qualitative, or mixed-methods (e.g. qualitative cross-sectional, quantitative experimental, mixed-methods case study).* |
| Research sample | *State the research sample (e.g. Harvard university undergraduates, villagers in rural India) and provide relevant demographic information (e.g. age, sex) and indicate whether the sample is representative. Provide a rationale for the study sample chosen. For studies involving existing datasets, please describe the dataset and source.* |
| Sampling strategy | *Describe the sampling procedure (e.g. random, snowball, stratified, convenience). Describe the statistical methods that were used to predetermine sample size OR if no sample-size calculation was performed, describe how sample sizes were chosen and provide a rationale for why these sample sizes are sufficient. For qualitative data, please indicate whether data saturation was considered, and what criteria were used to decide that no further sampling was needed.* |
| Data collection | *Provide details about the data collection procedure, including the instruments or devices used to record the data (e.g. pen and paper, computer, eye tracker, video or audio equipment) whether anyone was present besides the participant(s) and the researcher, and whether the researcher was blind to experimental condition and/or the study hypothesis during data collection.* |
| Timing | *Indicate the start and stop dates of data collection. If there is a gap between collection periods, state the dates for each sample cohort.* |
| Data exclusions | *If no data were excluded from the analyses, state so OR if data were excluded, provide the exact number of exclusions and the rationale behind them, indicating whether exclusion criteria were pre-established.* |
| Non-participation | *State how many participants dropped out/declined participation and the reason(s) given OR provide response rate OR state that no participants dropped out/declined participation.* |
| Randomization | *If participants were not allocated into experimental groups, state so OR describe how participants were allocated to groups, and if allocation was not random, describe how covariates were controlled.* |

# Ecological, evolutionary & environmental sciences study design

All studies must disclose on these points even when the disclosure is negative.

| | |
|---|---|
| Study description | *Briefly describe the study. For quantitative data include treatment factors and interactions, design structure (e.g. factorial, nested, hierarchical), nature and number of experimental units and replicates.* |
| Research sample | *Describe the research sample (e.g. a group of tagged Passer domesticus, all Stenocereus thurberi within Organ Pipe Cactus National Monument), and provide a rationale for the sample choice. When relevant, describe the organism taxa, source, sex, age range and any manipulations. State what population the sample is meant to represent when applicable. For studies involving existing datasets, describe the data and its source.* |
| Sampling strategy | *Note the sampling procedure. Describe the statistical methods that were used to predetermine sample size OR if no sample-size calculation was performed, describe how sample sizes were chosen and provide a rationale for why these sample sizes are sufficient.* |
| Data collection | *Describe the data collection procedure, including who recorded the data and how.* |
| Timing and spatial scale | *Indicate the start and stop dates of data collection, noting the frequency and periodicity of sampling and providing a rationale for these choices. If there is a gap between collection periods, state the dates for each sample cohort. Specify the spatial scale from which the data are taken* |
| Data exclusions | *If no data were excluded from the analyses, state so OR if data were excluded, describe the exclusions and the rationale behind them, indicating whether exclusion criteria were pre-established.* |
| Reproducibility | *Describe the measures taken to verify the reproducibility of experimental findings. For each experiment, note whether any attempts to repeat the experiment failed OR state that all attempts to repeat the experiment were successful.* |
| Randomization | *Describe how samples/organisms/participants were allocated into groups. If allocation was not random, describe how covariates were controlled. If this is not relevant to your study, explain why.* |
| Blinding | *Describe the extent of blinding used during data acquisition and analysis. If blinding was not possible, describe why OR explain why blinding was not relevant to your study.* |

Did the study involve field work? ☐ Yes ☒ No

# Reporting for specific materials, systems and methods

We require information from authors about some types of materials, experimental systems and methods used in many studies. Here, indicate whether each material, system or method listed is relevant to your study. If you are not sure if a list item applies to your research, read the appropriate section before selecting a response.

## Materials & experimental systems

| n/a | Involved in the study |
|---|---|
| ☐ | ☒ Antibodies |
| ☐ | ☒ Eukaryotic cell lines |
| ☒ | ☐ Palaeontology and archaeology |
| ☒ | ☐ Animals and other organisms |
| ☐ | ☒ Clinical data |
| ☒ | ☐ Dual use research of concern |
| ☒ | ☐ Plants |

## Methods

| n/a | Involved in the study |
|---|---|
| ☒ | ☐ ChIP-seq |
| ☐ | ☒ Flow cytometry |
| ☒ | ☐ MRI-based neuroimaging |

## Antibodies

Antibodies used

- Flow cytometry: CD3-APC/H7 (clone SK7; ref. 560176), CD4-AF700 (clone OKT4; ref. 56-0048-80; Invitrogen), CD8-BUV395 (clone RPA-T8; ref. 563796), CD25-APC (clone M-A251; ref. 561399), PD-1-BUV737 (clone EH12.1; ref. 612792) and CD69-PE (clone FN50; ref. 560968) (all BD Biosciences).
- T cell activation: anti-CD3 (0.1 µg/mL; clone OKT3; ref. 317325; Biolegend) and anti-CD28 (0.1 µg/mL; clone CD28.2; ref. 302933; Biolegend).
- Immunostaining: anti-PD-L1 (1:250; clone [28-8], ref. ab205921, Abcam), anti-PD1 (1:500; clone EPR4877(2), ref ab137132, Abcam), anti-PanCK (1:500, clone KRT/1877R, ref ab234297, Abcam), and anti-CD3 (1:500, polyclonal, ref. GA503, Agilent Dako).
- Pembrolizumab (5 mg; ref. A2005; Selleckchem).

Validation

- All the antibodies used in this study are well validated commercial products. They are routinely tested for the application selected and verified that bind to the antigen stated by the manufactures' company.
- For flow cytometry and immunostaining assays, negative and positive controls were included for each antibody.
- Pembrolizumab PD-1 receptor blocking efficiency was validated by flow cytometry and shown in Supplementary material.

## Eukaryotic cell lines

Policy information about cell lines and Sex and Gender in Research

| | |
|---|---|
| Cell line source(s) | MCF-7 and MDA-MB-231 breast cancer cell lines (ATCC; Manassas, VA, HTB-22TM and HBT-26TM respectively) |
| Authentication | None of the cell lines used were authenticated in-house. All of them were directly obtained from ATCC and authenticated by the manufacturers' company. |
| Mycoplasma contamination | All the cell lines tested negative for mycoplasma contamination. |
| Commonly misidentified lines (See ICLAC register) | N/A |

## Clinical data

Policy information about clinical studies

All manuscripts should comply with the ICMJE guidelines for publication of clinical research and a completed CONSORT checklist must be included with all submissions.

| | |
|---|---|
| Clinical trial registration | The study has clinical data but it is not a clinical trial. |
| Study protocol | It does not apply for this study. |
| Data collection | Biobanked tumors: Patients diagnosed with breast cancer from 1992 to 1999 and followed up until 2012.<br>Tumors collected directly after surgery: Patients diagnosed with breast cancer and undergoing for surgery between 2019-2022. |
| Outcomes | It does not apply for this study. |

## Plants

| | |
|---|---|
| Seed stocks | *Report on the source of all seed stocks or other plant material used. If applicable, state the seed stock centre and catalogue number. If plant specimens were collected from the field, describe the collection location, date and sampling procedures.* |
| Novel plant genotypes | *Describe the methods by which all novel plant genotypes were produced. This includes those generated by transgenic approaches, gene editing, chemical/radiation-based mutagenesis and hybridization. For transgenic lines, describe the transformation method, the number of independent lines analyzed and the generation upon which experiments were performed. For gene-edited lines, describe the editor used, the endogenous sequence targeted for editing, the targeting guide RNA sequence (if applicable) and how the editor was applied.* |
| Authentication | *Describe any authentication procedures for each seed stock used or novel genotype generated. Describe any experiments used to assess the effect of a mutation and, where applicable, how potential secondary effects (e.g. second site T-DNA insertions, mosiacism, off-target gene editing) were examined.* |

## Flow Cytometry

### Plots

Confirm that:

☒ The axis labels state the marker and fluorochrome used (e.g. CD4-FITC).

☒ The axis scales are clearly visible. Include numbers along axes only for bottom left plot of group (a 'group' is an analysis of identical markers).

☒ All plots are contour plots with outliers or pseudocolor plots.

☒ A numerical value for number of cells or percentage (with statistics) is provided.

### Methodology

| | |
|---|---|
| Sample preparation | T cells were Isolated using Dynabeads® Untouched™ Human T Cells kit (Invitrogen, Life Technologies) from PBMCs extracted from buffy coats. T cells were incubated with conjugated antibodies and flow cytometry analyses were performed on single cells after excluding dead cells with Live/Dead fixable aqua dead cell stain kit (Molecular Probes) |
| Instrument | LSRII flow cytometer (BD Biosciences) |
| Software | FACS Diva software (BD Biosciences) and FlowJo software (TreeStar Inc) |
| Cell population abundance | At least 10 000 alive single cells were analyzed where 98-95% were CD3+ |
| Gating strategy | 1. Exclude cell debris and other cell types: SSC-A/FSC-A.<br>2. Exclude doublets. Single cell gate: FSC-H/FSC-A.<br>3. Select alive cells expression CD3+: CD3-APC-H7/BV510-A (live/dead stain). |

☒ Tick this box to confirm that a figure exemplifying the gating strategy is provided in the Supplementary Information.
